# Supplementary material for: Trade-off between tree planting and wetland conservation in China
Source: Nat Commun. 2022 Apr 12;13:1967. doi: 10.1038/s41467-022-29616-7 (PMC9005732; doi:10.1038/s41467-022-29616-7)
Supplement: Supplementary file 1 — Supplementary Information [file 41467_2022_29616_MOESM1_ESM.pdf]

Supplementary Information for
Trade-off between tree planting and wetland conservation in China

Yi Xi[1], Shushi Peng[1*], Gang Liu[1], Agnès Ducharne[2], Philippe Ciais[3,4],

Catherine Prigent[5,6], Xinyu Li[1], Xutao Tang[1]

[1]Sino-French Institute for Earth System Science, College of Urban and Environmental Sciences, and Laboratory for Earth Surface Processes, Peking University, Beijing, China

[2]Sorbonne Université, CNRS, EPHE, Laboratoire METIS (Milieux environnementaux, transferts et interaction dans les hydrosystèmes et les sols), 75005 Paris, France

[3]Laboratoire des Sciences du Climat et de l'Environnement, LSCE/IPSL, CEA-CNRS-UVSQ, Université Paris-Saclay 91191 Gif-sur-Yvette, France

[4]The Cyprus Institute 20 Konstantinou Kavafi Street, 2121, Nicosia, Cyprus

[5]CNRS, Sorbonne Université, Observatoire de Paris, Université PSL, LERMA, Paris, France

[6]Estellus, Paris, France

[*]Correspondence to Shushi Peng (speng@pku.edu.cn)

Contents of this file

Supplementary Text 1 and 2

Supplementary Tables 1 to 3

Supplementary Figures 1 to 19

Supplementary Text

Supplementary Text 1. Uncertainty of inventory-based forest data.

To validate the spatial distributions and temporal variations in forest cover fraction from our inventory-based forest maps, we used two satellite-based forest cover fraction data, SXP-VCF[1] and MOD44B-VCF (Supplementary Table 3). Generally, due to different forest definitions, spatial resolution, and acquisition algorithms[2], the spatial patterns and temporal trends of forest coverage show substantial discrepancies among different forest cover data. For the absolute forest area, although almost all forest data consistently show a higher forest cover fraction in southern and northeastern China, the two satellite-based forest cover data report a higher or lower forest cover fraction (~10–20%) at 0.5° × 0.5° resolution compared with our inventory-based data (Supplementary Fig. 18). The mean annual forest area from SXP-VCF, MOD44B-VCF, and our forest maps for 2000–2016 are 1.42 Mkm^2, 1.63 Mkm^2, 1.60 Mkm^2, respectively. While for the temporal trends, SXP-VCF and MOD44B-VCF show a good agreement in spatial patterns and absolute values of the trend in forest coverage with our simulation, suggesting a > 0.3% yr^{-1} increase in forest cover fraction in central and southwestern regions of China from 2000 to 2016 (Supplementary Fig. 19). Besides, the two forest data consistently present an increasing forest coverage but a decreasing wetland extent in >50% grids in the absence of human disturbance, suggesting high confidence of forest change derived from the national forest inventory.

Supplementary Text 2. Algorithm to produce the annual land-cover maps.

For the historical land-cover maps, we combined the spatial information from the 1:1,000,000 Chinese Vegetation Map[3] and annual forest change linearly interpolated from the five-year-interval forest inventory data. Since ORCHIDEE-Hillslope needs land cover maps with 12 PFTs (including 9 forest types) while the static Chinese Vegetation Map is given at the vegetation formation/sub-formation level, we first grouped the plant species in the Vegetation Map into the 12 PFTs to match with the ORCHIDEE-Hillslope according to their climate regions, vegetation phenology type, and physiognomy based on the knowledge of Flora of China (http://frps.eflora.cn/). The shapefile of the PFT map was then resampled into 0.1° × 0.1° resolution and aggregated to compute the fractional cover (%) of each PFT at a spatial resolution of 0.5° × 0.5°. Given that the field surveys to compile the Vegetation Map was done in the 1980s, the 0.5° × 0.5° map was regarded as the fundamental vegetation map in 1979, corresponding to the second forest inventory (1977–1981). After that, the total forest change at the province level was proportionally allocated to the forest grid cells in the province according to their forest cover fraction in the fundamental vegetation map, and then the fraction of other vegetation types including bare soil, grasslands, and croplands defined by ORCHIDEE-Hillslope changed proportionally.

For near-term land-cover maps, the goal of covering 26% of China from the national 15-year Comprehensive Plan is for arbor forests, bamboo forests and shrubs. We assumed a business-as-usual tree planting scenario which allows the area of arbor forests to increase at the same rate as under the historical scenario. As a result, the total area of arbor forests is projected to cover 2.28 Mkm^2, accounting for 24.0% of the country by 2035. For S_A, we first added the annual forest increment to the historical land cover map in 2016 at grid-scale. When the increase of forest area (denoted by A_f) is larger than the available area in the grid cell (denoted by A_{gc}), the excess forest expansion ($A_f - A_{gc}$) was proportionally distributed to the grids within

its nearest 3 × 3 or broader neighbourhood, and vice versa. Then the fractions of other vegetation types changed proportionally. The algorithm was chosed for simplicity in this study, which could be unrealistic due to the lack of the consideration of the possibility of climate, edaphic, and topographic conditions for tree planting and urban development. To evaluate the effects of different locations of tree planting on China's wetland conservation, we additionally designed three extreme scenarios to plant all new forests under S_A into climate zones of PET/P > 2 (S_A^{dry}), PET/P of 1–2 (S_A^{mesic}), and PET/P < 1 (S_A^{wet}) (Fig. 5b–d). The distribution of new forests to each grid cell was treated with the same processes as S_A.

Supplementary Tables

Supplementary Table 1. Simulated protocols of climate change, CO_2, and land cover change using ORCHIDEE-Hillslope.

Scenario	Simulations	Period	Climate	CO_2	Land Cover
Historical Simulations					
Spin-up	S0	1800–1999	Cycled GSWP3-W5E5 1980–1999	2000	2000
Historical	S0	2000–2016	GSWP3-W5E5 2000–2016	2000–2016	2000
	S1	2000–2016	GSWP3-W5E5 2000–2016	2000–2016	2000–2016
Near-term simulations with constant climate as in 2000–2016					
S_A	S0	2017–2035	Random GSWP3-W5E5 2000–2016[a]	2016	2016
	S1	2017–2035	Random GSWP3-W5E5 2000–2016[a]	2016	2017–2035
S_A^{dry}	S0	2017–2035	Random GSWP3-W5E5 2000–2016[a]	2016	2016
	S1	2017–2035	Random GSWP3-W5E5 2000–2016[a]	2016	2017–2035 (dry)
S_A^{mesic}	S0	2017–2035	Random GSWP3-W5E5 2000–2016[a]	2016	2016
	S1	2017–2035	Random GSWP3-W5E5 2000–2016[a]	2016	2017–2035 (mesic)
S_A^{wet}	S0	2017–2035	Random GSWP3-W5E5 2000–2016[a]	2016	2016
	S1	2017–2035	Random GSWP3-W5E5 2000–2016[a]	2016	2017–2035 (wet)
Near-term simulations with future climate from ISIMIP3b					
Spin-up	S0	1800–1999	Cycled ISIMIP3b 1980–1999[b]	2000	2000
Transient	S1	2000–2014	ISIMIP3b 2000–2014[b]	2000–2014	2000–2014
		2015–2016	ISIMIP3b 2015–2016[c]	2015–2016	2015–2016
S_B	S0	2017–2035	ISIMIP3b 2017–2035[c]	2017–2035	2016
	S1	2017–2035	ISIMIP3b 2017–2035[c]	2017–2035	2017–2035

[a] The four groups of near-term simulations for S_A are performed with a 19-year climate forcing randomly generated from GSWP3-W5E5. The 19 random years comprise 2008, 2003, 2014, 2002, 2001, 2009, 2012, 2005, 2003, 2005, 2003, 2008, 2015, 2010, 2002, 2006, 2001, 2008, and 2007.

[b] The Spin-up for 1800–1999 and the Transient simulations for 2000–2014 use historical climate forcing from ISIMIP3b. The historical scenario from ISIMIP3b includes five climate models (GFDL-ESM4, IPSL-CM6A-LR, MPI-ESM1-2-HR, MRI-ESM2-0, and UKESM1-0-LL), thus the Spin-up simulations are performed for each climate model respectively.

[c] The Transient simulations for 2015–2016 and S_B for 2017–2035 use future climate forcing from ISIMIP3b, which includes three SSPs (SSP1-2.6, SSP3-7.0, and SSP5-8.5), with five models (GFDL-ESM4, IPSL-

CM6A-LR, MPI-ESM1-2-HR, MRI-ESM2-0, and UKESM1-0-LL) in each SSP.

Supplementary Table 2. Projected impacts of Climate Change (CC), elevated CO_2 (eCO_2), and forest change on wetland change for 2017–2035 under the S_B scenario. The relative impact of forest change on wetland change to all three factors is derived by dividing the trend in wetland change due to CC, eCO_2, and forest change (S1) by the trend in wetland change due to forest change (S1 minus S0). The first values indicate the multi-model mean trend in wetland change, while superscripts and subscripts after mean values indicate the maximum and minimum values. The trends are estimated by linear least-squares regression. The black and bold values indicate the trend is statistically significant (*t*-test, $p < 0.05$) while the insignificant trends are in grey (*t*-test, $p > 0.05$).

Regions	Factors	SSP1-2.6	SSP3-7.0	SSP5-8.5
China	CC + eCO_2 + forest change ($km^2\ yr^{-1}$)	-298^{1074}_{-2375}	82^{2086}_{-1845}	-188^{2137}_{-1949}
	CC + eCO_2 ($km^2\ yr^{-1}$)	-229^{1137}_{-2303}	157^{2144}_{-1753}	-111^{2220}_{-1872}
	forest change ($km^2\ yr^{-1}$)	-69^{-64}_{-77}	-75^{-58}_{-92}	-77^{-67}_{-83}
	Relative impact of forest change (%)	23^{11}_{-125}	-92^{8}_{-13}	41^{28}_{-4}
PET/P > 2	CC + eCO_2 + forest change ($km^2\ yr^{-1}$)	-73^{356}_{-1107}	-14^{667}_{-556}	-125^{428}_{-903}
	CC + eCO_2 ($km^2\ yr^{-1}$)	-42^{386}_{-1078}	16^{693}_{-523}	-92^{464}_{-873}
	forest change ($km^2\ yr^{-1}$)	-31^{-29}_{-34}	-31^{-26}_{-34}	-33^{-30}_{-36}
	Relative impact of forest change (%)	43^{36}_{-22}	215^{43}_{-28}	26^{5}_{-27}
1 < PET/P < 2	CC + eCO_2 + forest change ($km^2\ yr^{-1}$)	-59^{640}_{-1151}	93^{938}_{-865}	-147^{967}_{-1012}
	CC + eCO_2 ($km^2\ yr^{-1}$)	-23^{667}_{-1111}	131^{966}_{-814}	-108^{1008}_{-965}
	forest change ($km^2\ yr^{-1}$)	-36^{-27}_{-41}	-38^{-28}_{-51}	-39^{-30}_{-46}
	Relative impact of forest change (%)	61^{10}_{-38}	-41^{22}_{-12}	26^{51}_{-4}
PET/P < 1	CC + eCO_2 + forest change ($km^2\ yr^{-1}$)	-38^{124}_{-174}	-25^{117}_{-159}	-38^{271}_{-296}
	CC + eCO_2 ($km^2\ yr^{-1}$)	-33^{128}_{-166}	-20^{121}_{-158}	-32^{278}_{-287}
	forest change ($km^2\ yr^{-1}$)	-5^{-3}_{-8}	-4^{-2}_{-8}	-6^{-2}_{-9}
	Relative impact of forest change (%)	13^{432}_{-4}	18^{6}_{-23}	16^{5}_{-5}
Protected basins	CC + eCO_2 + forest change ($km^2\ yr^{-1}$)	-37^{939}_{-1285}	80^{1122}_{-696}	-7^{1205}_{-949}
	CC + eCO_2 ($km^2\ yr^{-1}$)	0^{971}_{-1247}	119^{1148}_{-644}	32^{1248}_{-907}
	forest change ($km^2\ yr^{-1}$)	-37^{-32}_{-41}	-39^{-26}_{-52}	-40^{-33}_{-44}
	Relative impact of forest change (%)	99^{41}_{-8}	-48^{7}_{-15}	543^{418}_{-4}

BAS_N	CC + eCO_2 + forest change ($km^2\ yr^{-1}$)	$68_{\mathbf{-508}}^{310}$	$32_{-228}^{\mathbf{386}}$	-3_{-214}^{193}
	CC + eCO_2 ($km^2\ yr^{-1}$)	$81_{\mathbf{-493}}^{320}$	$47_{-214}^{\mathbf{395}}$	11_{-198}^{210}
	forest change ($km^2\ yr^{-1}$)	$\mathbf{-13_{-15}^{-10}}$	$\mathbf{-15_{-21}^{-10}}$	$\mathbf{-14_{-18}^{-9}}$
	Relative impact of forest change (%)	-19_{-5}^{94}	-47_{-20}^{9}	514_{-43}^{29}
BAS_P	CC + eCO_2 + forest change ($km^2\ yr^{-1}$)	$-46_{\mathbf{-578}}^{402}$	$39_{-425}^{\mathbf{490}}$	61_{-427}^{521}
	CC + eCO_2 ($km^2\ yr^{-1}$)	$-33_{\mathbf{-566}}^{\mathbf{413}}$	$53_{-407}^{\mathbf{501}}$	$75_{-411}^{\mathbf{538}}$
	forest change ($km^2\ yr^{-1}$)	$\mathbf{-13_{-15}^{-12}}$	$\mathbf{-14_{-18}^{-11}}$	$\mathbf{-15_{-17}^{-11}}$
	Relative impact of forest change (%)	29_{-14}^{52}	-37_{-11}^{6}	-24_{-197}^{4}
BAS_{MC}	CC + eCO_2 + forest change ($km^2\ yr^{-1}$)	-67_{-314}^{206}	-35_{-293}^{176}	$-80_{\mathbf{-371}}^{329}$
	CC + eCO_2 ($km^2\ yr^{-1}$)	-56_{-304}^{216}	-25_{-279}^{182}	-70_{-362}^{338}
	forest change ($km^2\ yr^{-1}$)	$\mathbf{-11_{-16}^{-7}}$	$\mathbf{-9_{-14}^{-6}}$	$\mathbf{-10_{-13}^{-8}}$
	Relative impact of forest change (%)	16_{-265}^{42}	27_{-18}^{5}	13_{-63}^{9}

Supplementary Table 3. List of forest data used in this study.

Name and reference	Period	Spatial resolution	Forest definition	Source
NFI (National Forest Inventory)	1973–2018	Provincial	Tree cover ⩾ 20%	China's State Forestry Administration
SXP-VCF (Song et al. (ref. [1]))	1982–2016	0.05°	Canopy > 5 m	https://lpdaac.usgs.gov/products/vcf5kyrv001/#tools
MOD44B-VCF	2000–2019	250 m	Canopy > 5 m	https://lpdaac.usgs.gov/products/mod44bv006/

Supplementary Figures

Supplementary Figure 1. Map of nine regions in China. The regions used to describe the forest change or wetland change in the main text are as given by this map.

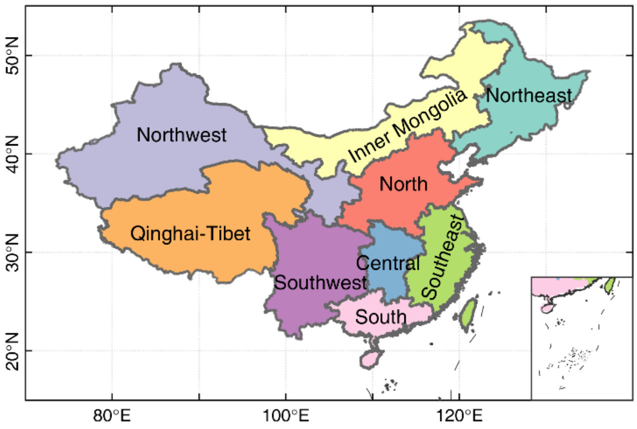

Supplementary Figure 2. Budyko curves with three different values of the parameter w according to equations (2) and (3). Figs. (**a**) and (**b**) represent the relationship between the ratio of evapotranspiration (ET) to precipitation (P), (ET/P) and the ratio of potential evapotranspiration (PET) to precipitation (P), (PET/P) following equation (2), while Figs. (**c**) and (**d**) represent the relationship between the ratio of runoff (Q) to precipitation (P), (Q/P) and PET/P following equation (3). The blue, green, and red curves describe the Budyko relationships when the parameter w is equal to 0.5 (grassland), 1, and 2 (forest), respectively. The dark grey points indicate the simulated ET/P versus PET/P for 2000–2016 in China at grid (**a**) and basin (**b**) scale from ORCHIDEE-Hillslope.

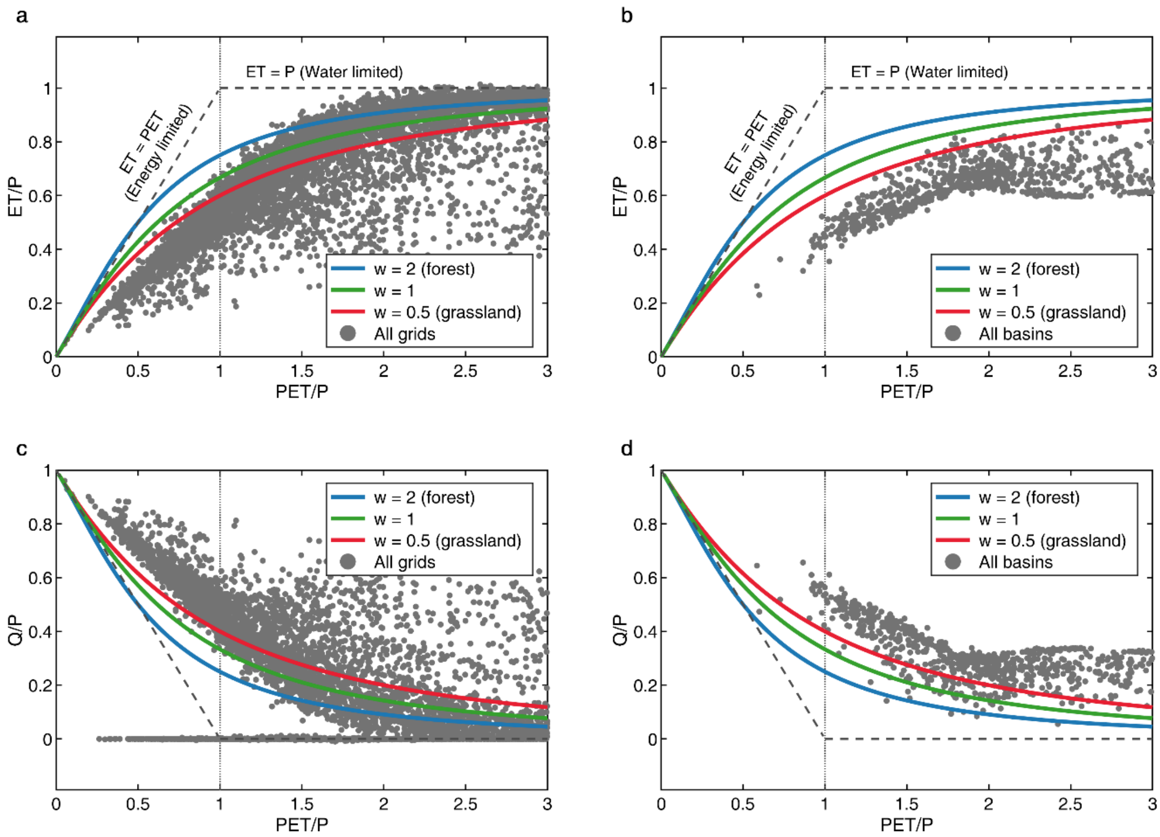

Supplementary Figure 3. Same as Fig. 2 (**a**)–(**d**), but for catchment scale.

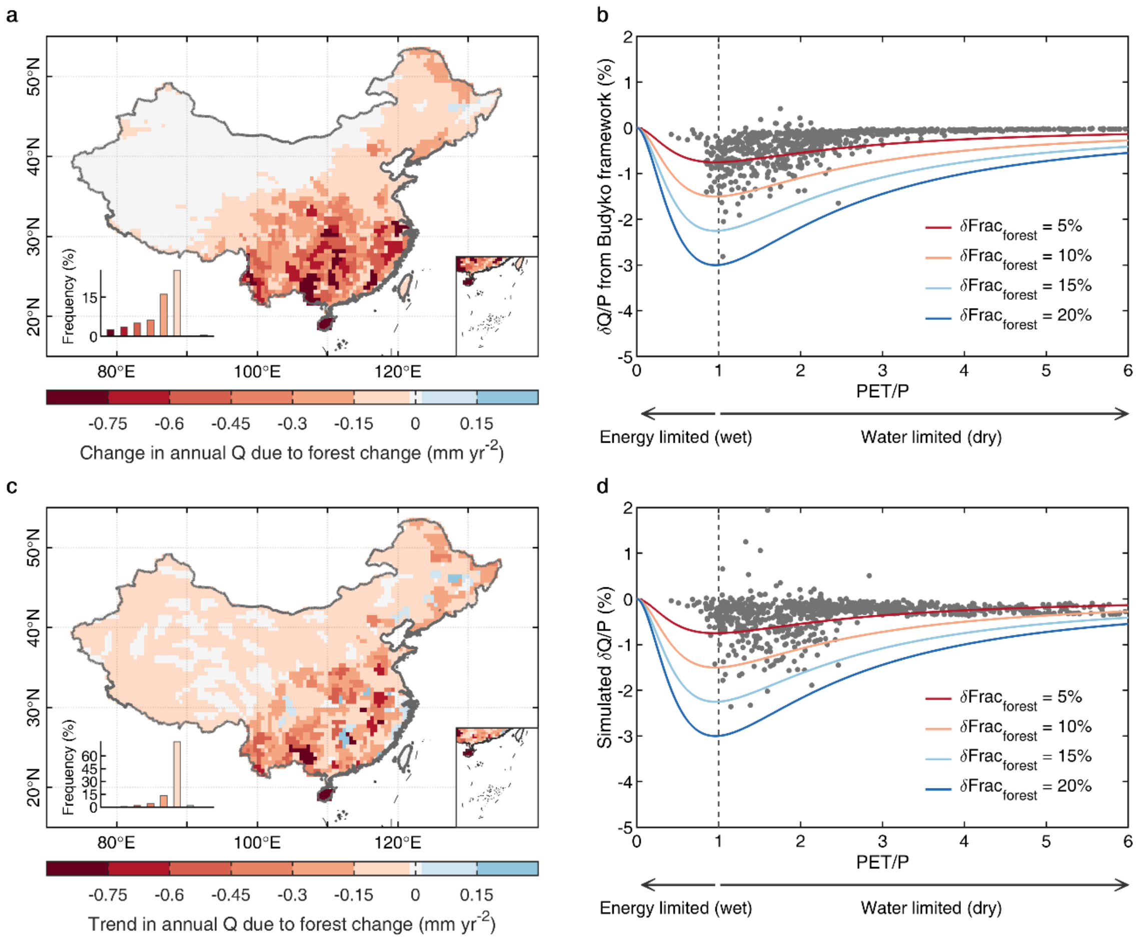

Supplementary Figure 4. Spatial patterns of mean annual precipitation (P), potential evapotranspiration (PET), and the ratio of potential evapotranspiration (PET) to precipitation (P), (PET/P) from 2000 to 2016 from GSWP3-W5E5. The PET is output directly from ORCHIDEE-Hillslope.

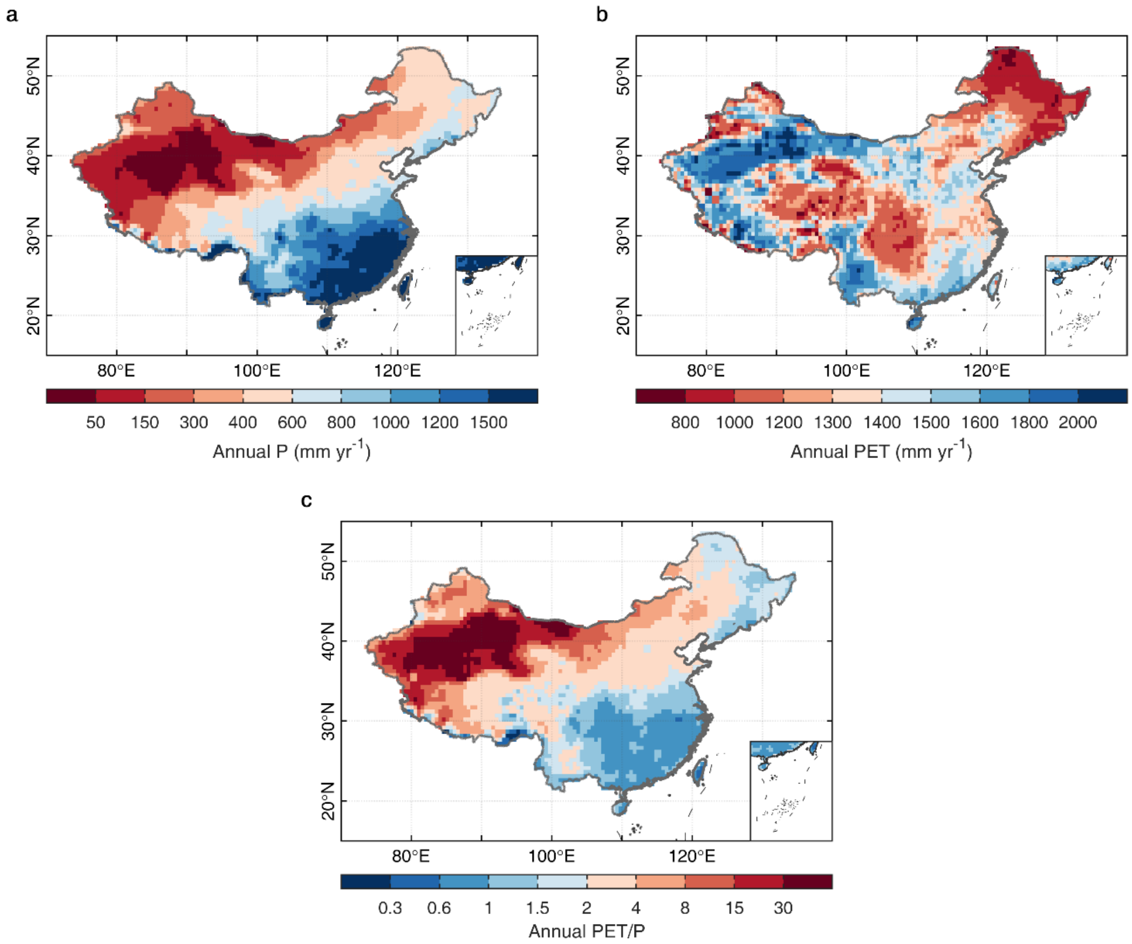

Supplementary Figure 5. Spatial patterns of long-term maximum wetland area from RFW (**a**), annual maximum wetland area from GIEMS-2 for 2000–2016 (**b**), as well as corresponding simulations (**c-d**) and RMSE of wetland area between RFW/GIEMS-2 and our simulations (**e-f**).

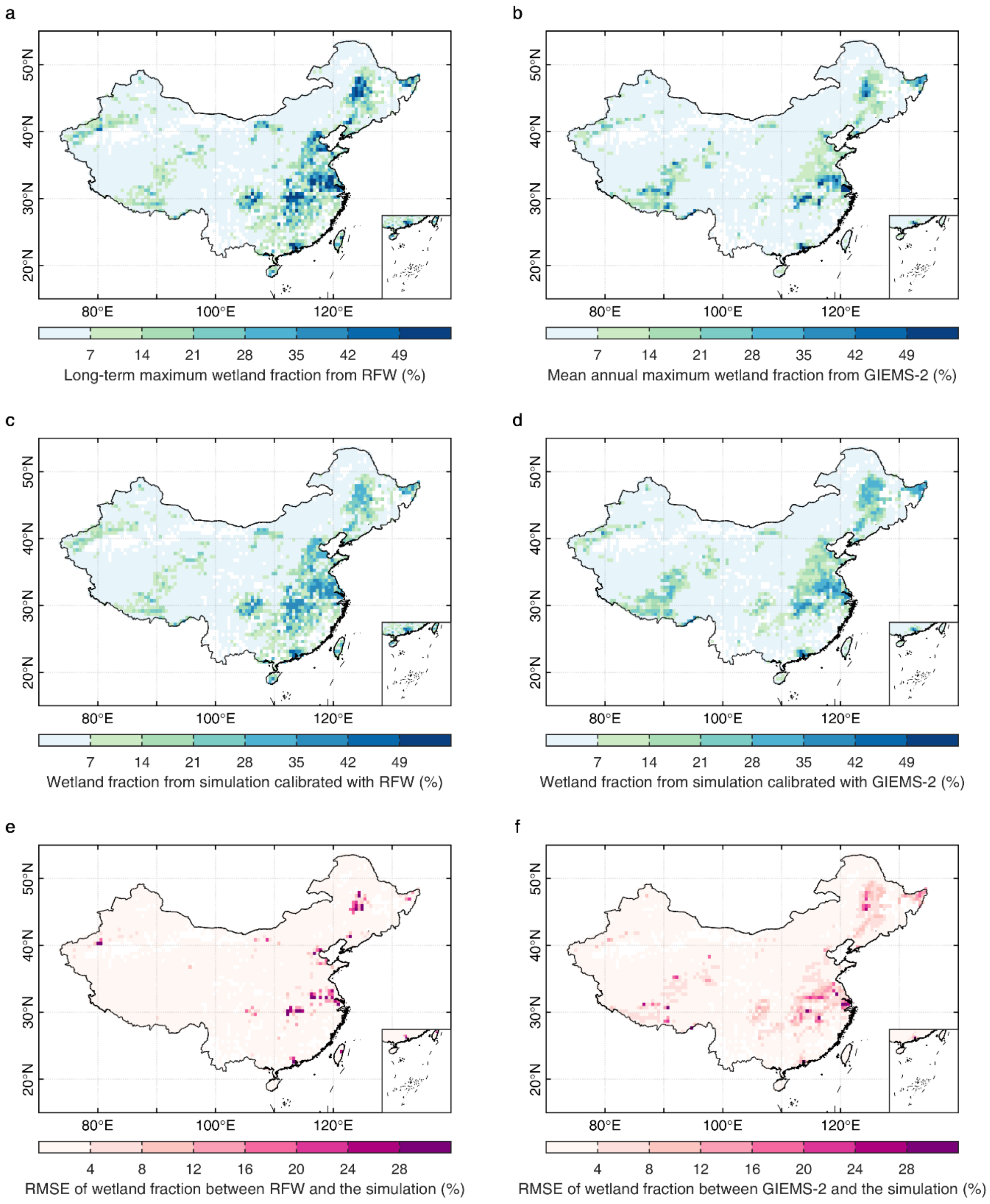

Supplementary Figure 6. Interannual variabilities of annual maximum wetland area (**a**) and annual maximum wetland area anomaly (**b**) from 2000 to 2016 from GIEMS-2 and the simulations calibrated with RFW and GIEMS-2.

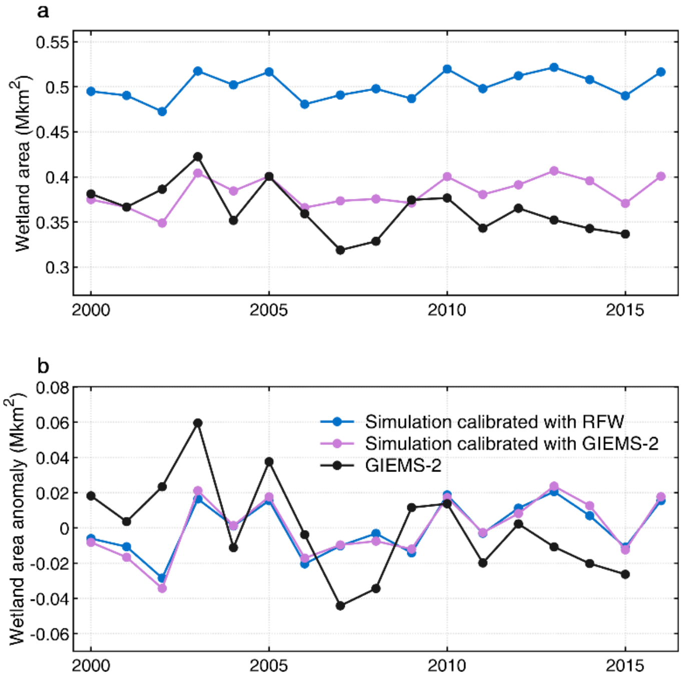

Supplementary Figure 7. Same as Fig. 3b, but for spatial patterns of the sensitivity of wetland to SM change ($\frac{\delta A_{wet}}{\delta SM}$) (**a**) and the sensitivity of SM to forest change ($\frac{\delta SM}{\delta A_{forest}}$) from 2000 to 2016 simulated with ORCHIDEE-Hillslope.

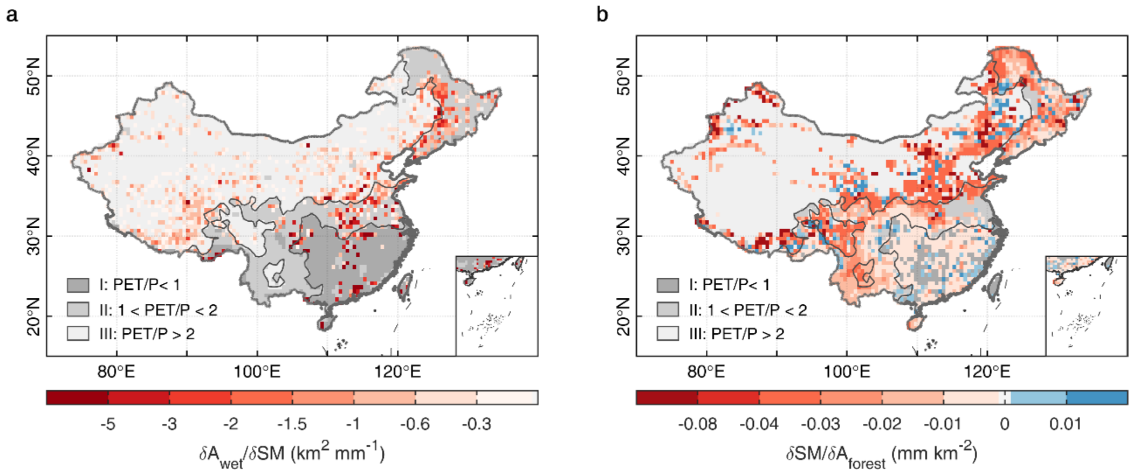

Supplementary Figure 8. Importance of basins containing protected wetlands. (**a**) and (**b**), Spatial patterns of long-term maximum wetland fraction from RFW and annual maximum wetland fraction for 2000–2015 from GIEMS-2 across protected basins. (**c**), Spatial patterns of forest cover fraction from our inventory-based forest map across protected basins. (**d**) and (**e**), Spatial patterns of protected types of wetlands in the basins with different levels of wetland conservation. Only basins containing wetlands of the corresponding protected type are coloured. (**e**), Statistics of protected types of basins with different levels of wetland conservation. T1 and T2 indicate protected type 1 and protected type 2, respectively.

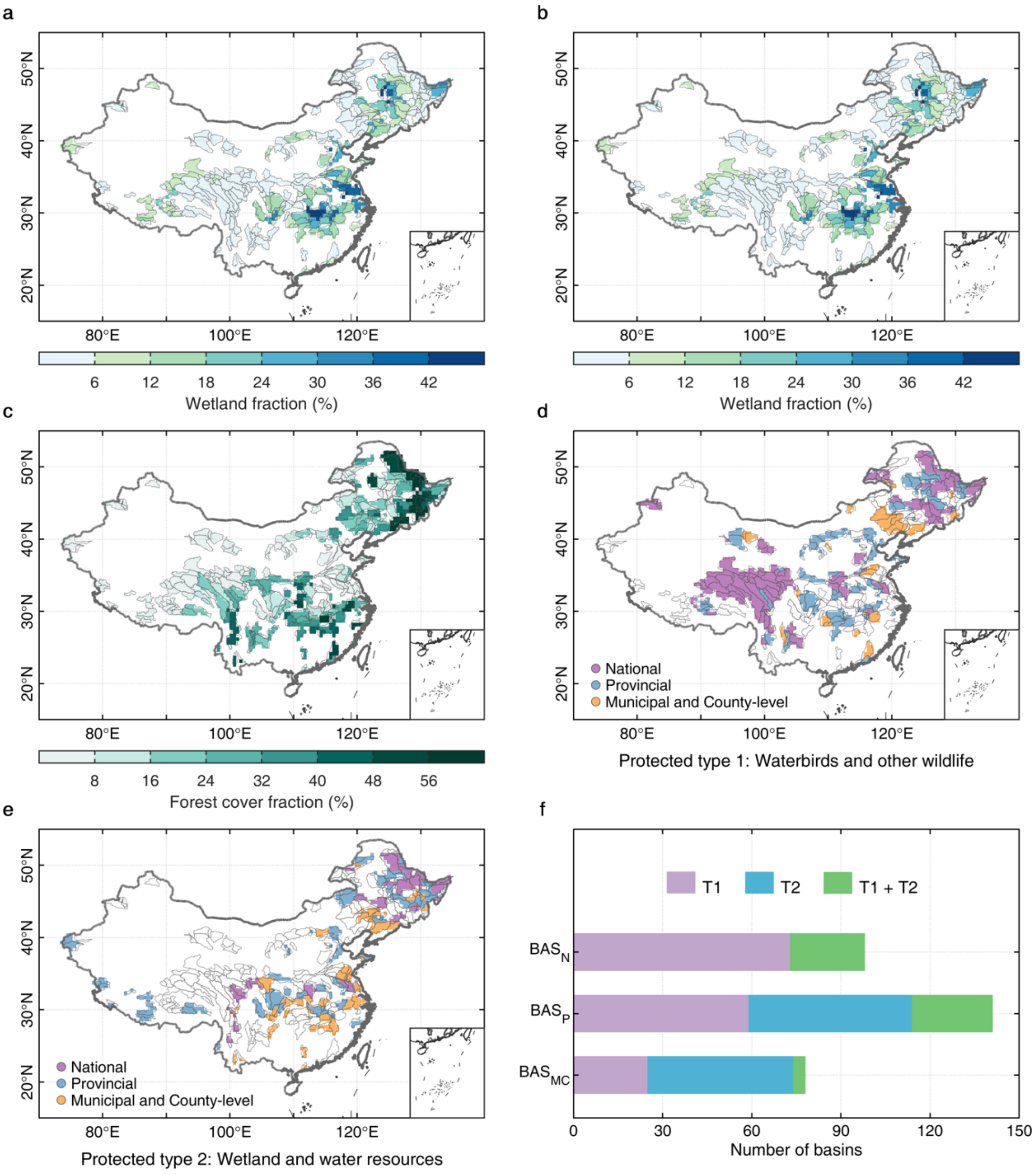

Supplementary Figure 9. Boundary of tree planting for 10% wetland loss for protected basins. (**a**) and (**c**), Spatial patterns of the potential forest cover fraction for 10% wetland loss (F_{forest} potential) at basin scale and at grid scale, inferred from historical wetland sensitivity to forest change (i.e., 10% of baseline wetland area divided by wetland sensitivity equals F_{forest} potential). (**b**) and (**d**), Spatial patterns of the difference between the forest change (δF_{forest}) under historical scenario and F_{forest} potential at basin scale and at grid scale. The difference can approximately represent the risk of wetland loss.

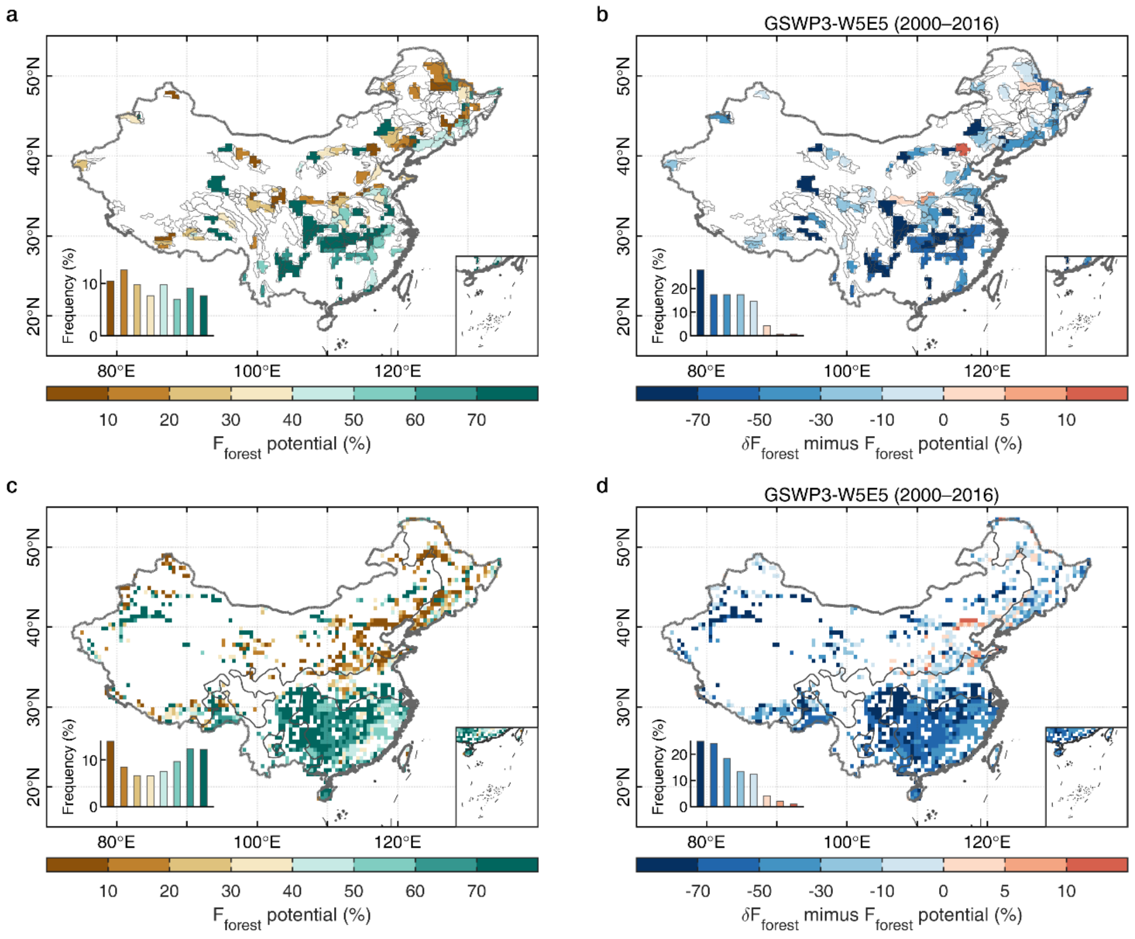

Supplementary Figure 10. Spatial patterns of forest change in China under S_A (**a**), S_A^{dry} (**b**), S_A^{mesic} (**c**), and S_A^{wet} (**d**) respectively.

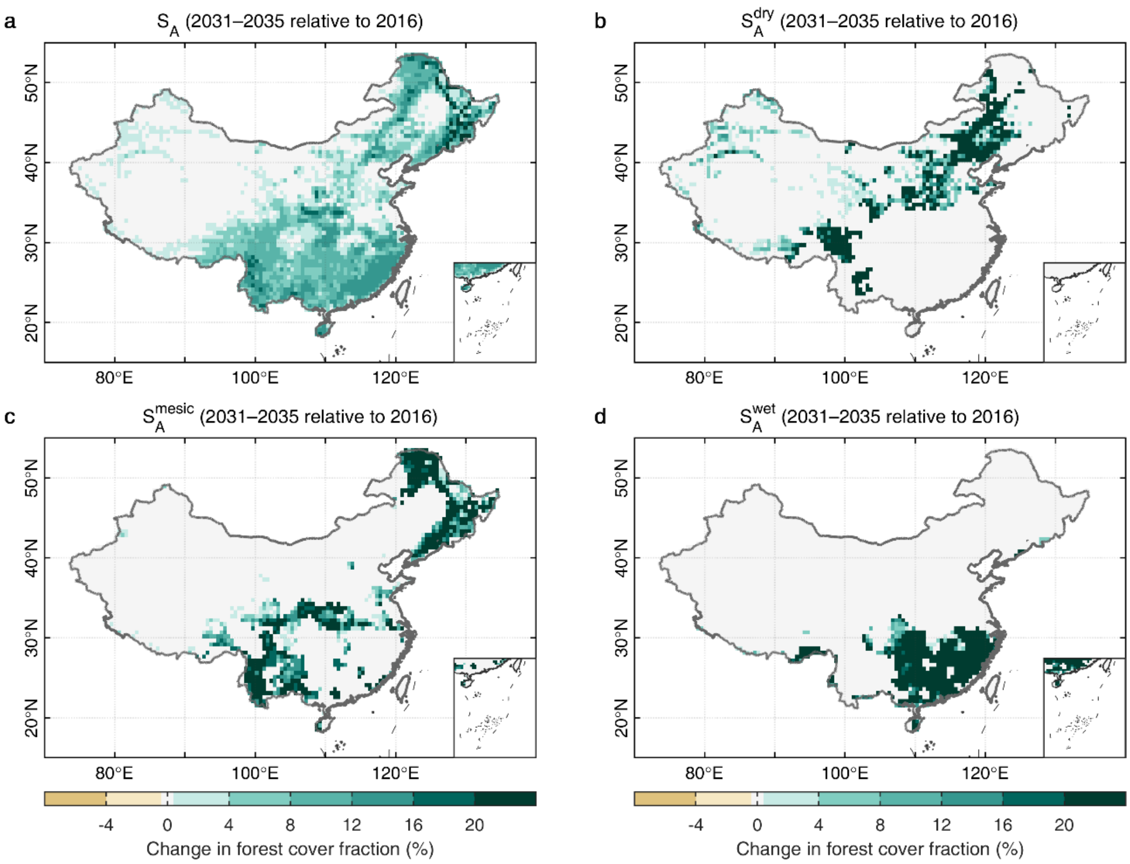

Supplementary Figure 11. Forest change and projected wetland change due to forest change across all grids, wetland grids, protected basins, and protected wetland grids under historical scenario (**a**), S_A (**b**), S_A^{dry} (**c**), S_A^{mesic} (**d**), and S_A^{wet} (**e**) by three climate zones.

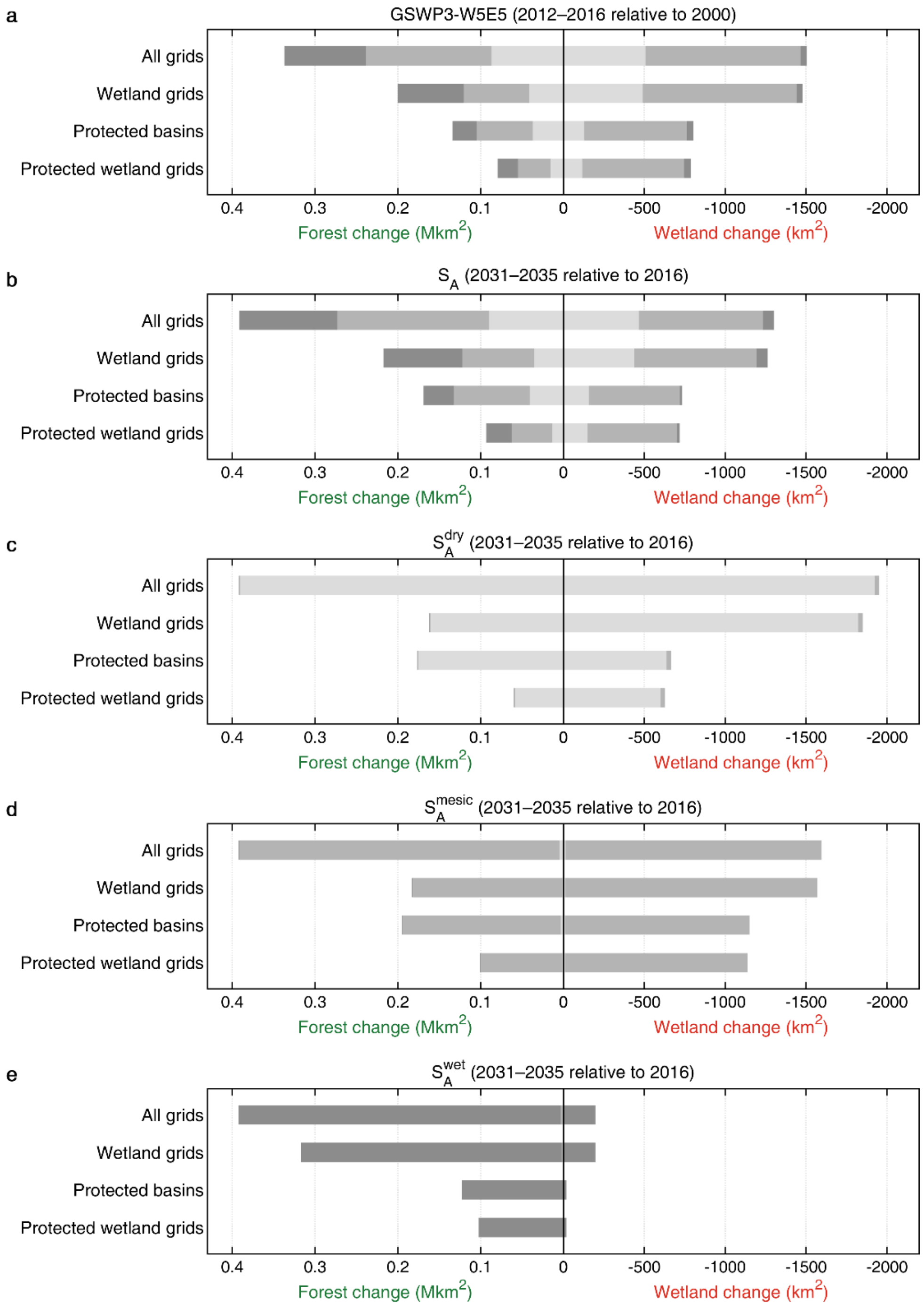

Supplementary Figure 12. Temporal change of near-term climate for 2017–2035 under three SSPs from ISIMIP3b. (**a**), Temperature anomaly, (**b**), Precipitation anomaly, and (**c**) Shortwave radiation anomaly relative to 2017–2035 (five-year moving average). The solid lines show the multi-model mean projections, and the shading presents the likely ranges estimated from the five individual climate models. The time means for 2031–2035 are shown as coloured vertical bars.

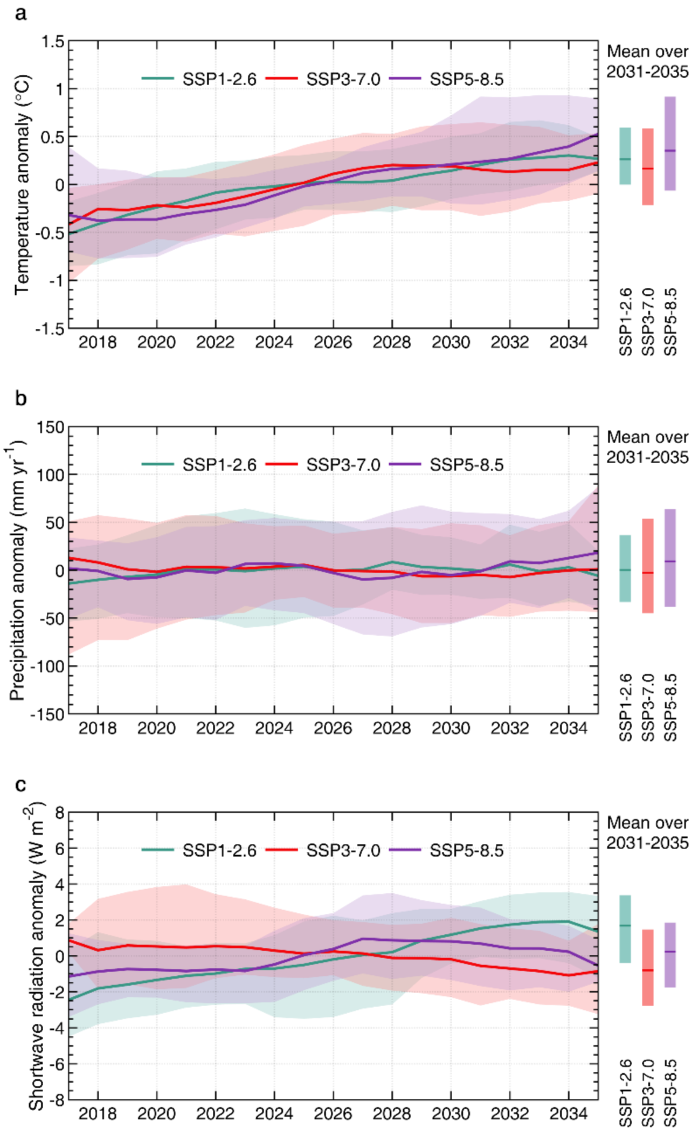

Supplementary Figure 13. Spatial patterns of trend in annual precipitation from the multi-model mean projections for 2017–2035 under three scenarios from ISIMIP3b. (**a**), SSP1-2.6; (**b**) SSP3-7.0; and (**c**) SSP5-8.5. The trends are estimated by linear least-squares regression and hatching indicates the trend is statistically significant (*t*-test, $p < 0.05$).

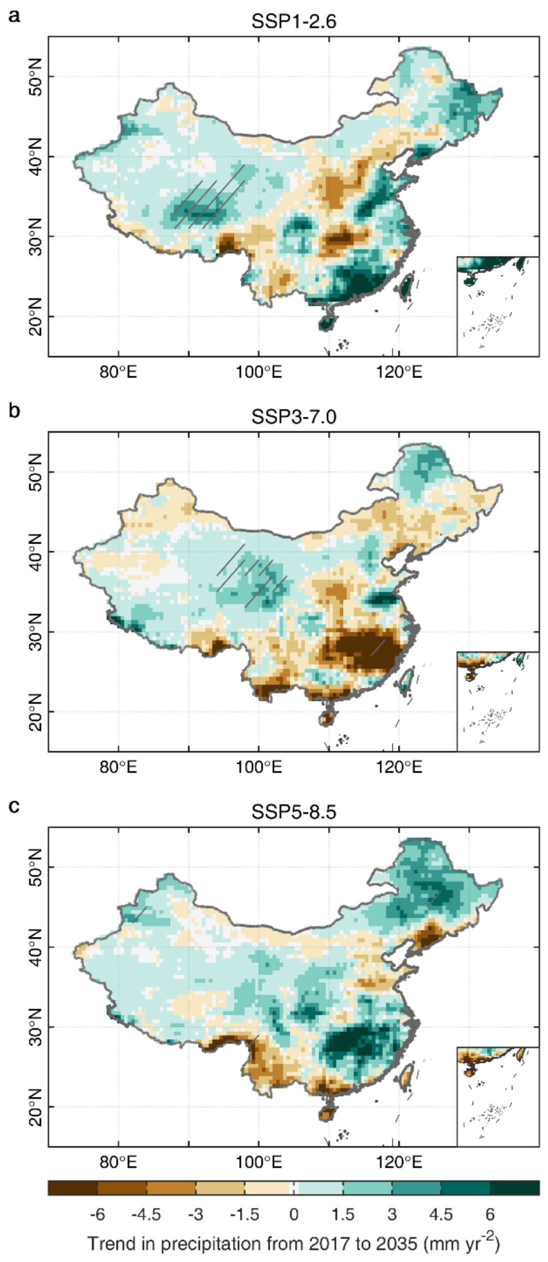

Supplementary Figure 14. Same as Fig. 3, but the wetland model calibrated with GIEMS-2. n = 275, n = 271, and n = 256 for three climate zones, respectively in Figs. (**c**)–(**e**).

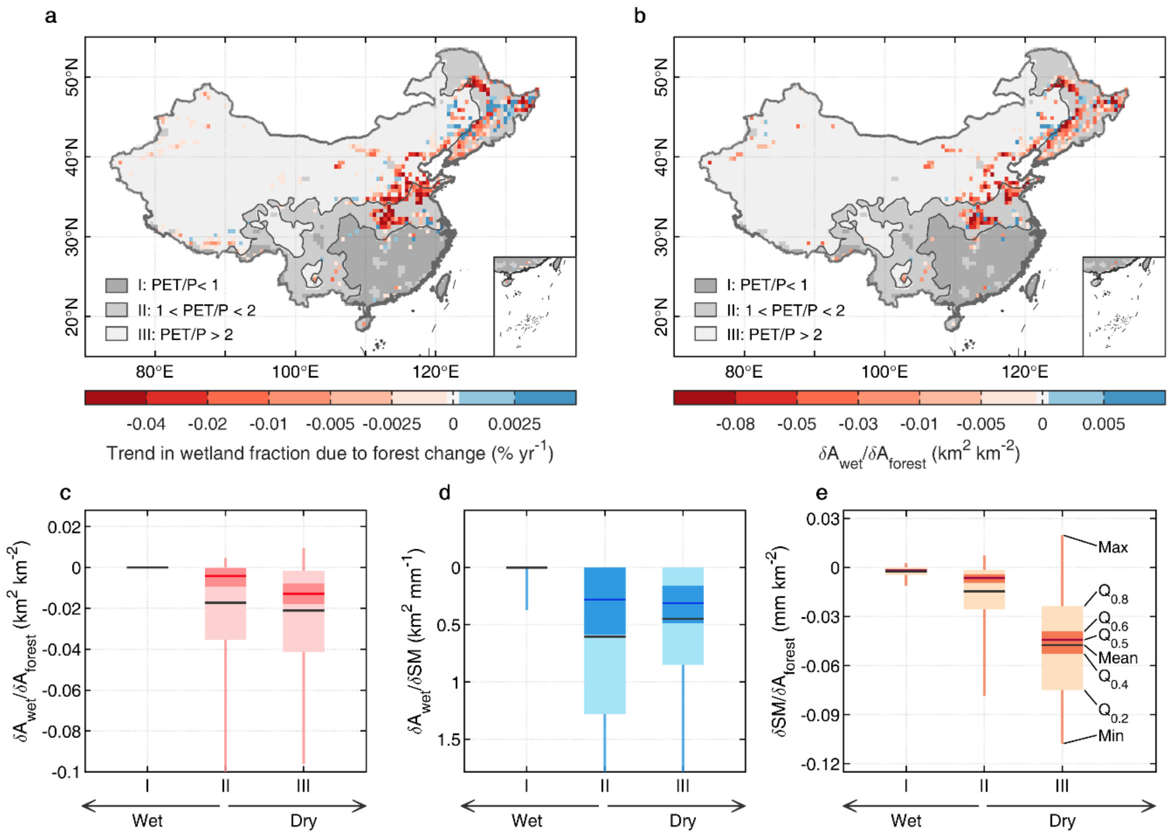

Supplementary Figure 15. Same as Fig. 4, but the wetland model calibrated with GIEMS-2.

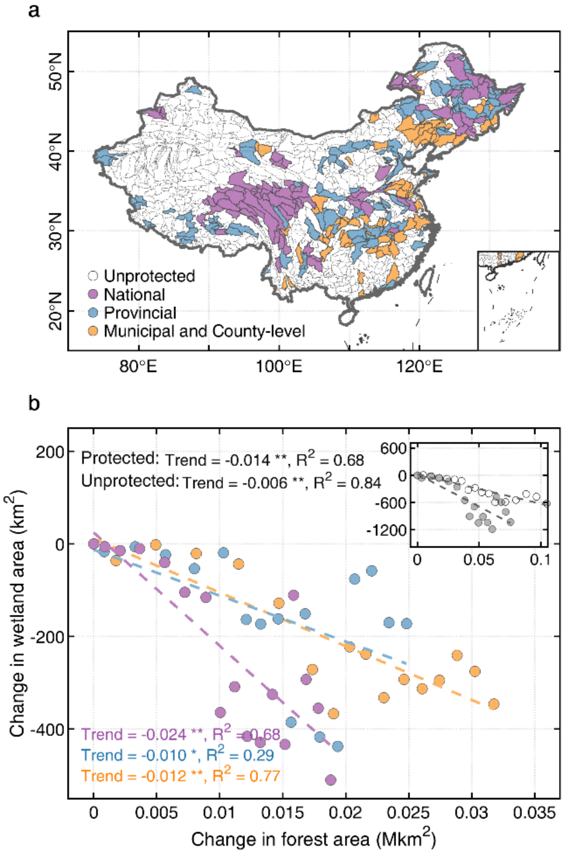

Supplementary Figure 16. Same as Fig. 5, but the wetland model calibrated with GIEMS-2.

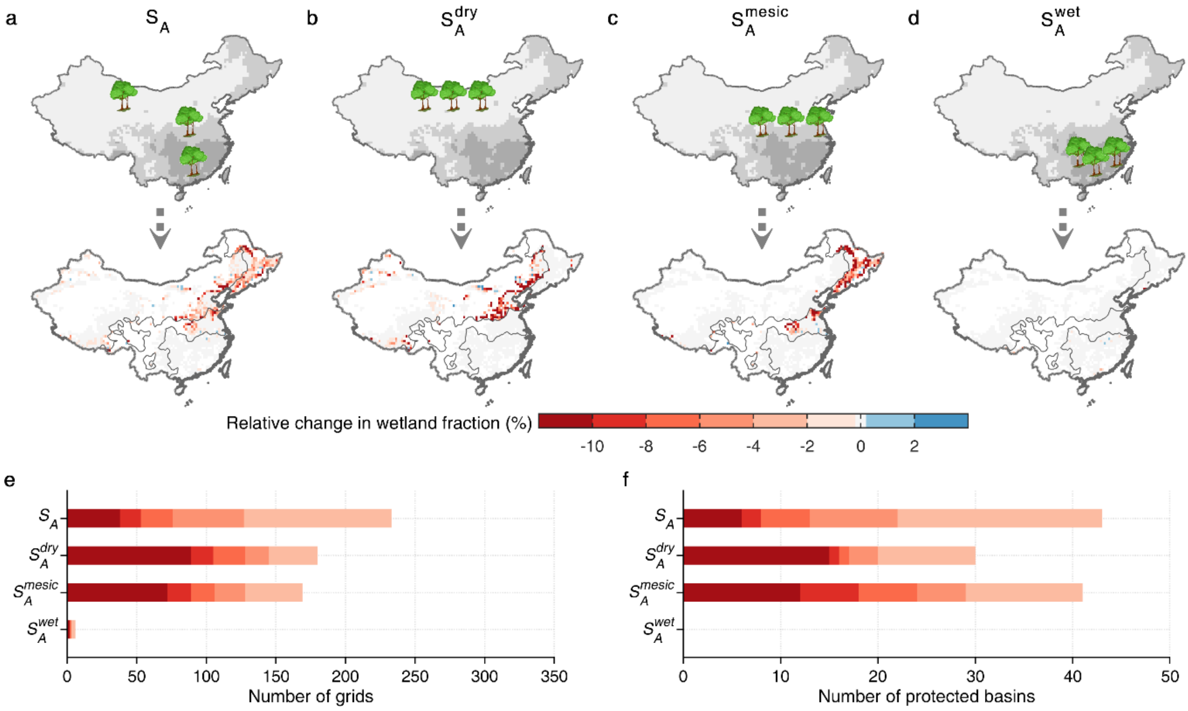

Supplementary Figure 17. Same as Fig. 6, but the wetland model calibrated with GIEMS-2.

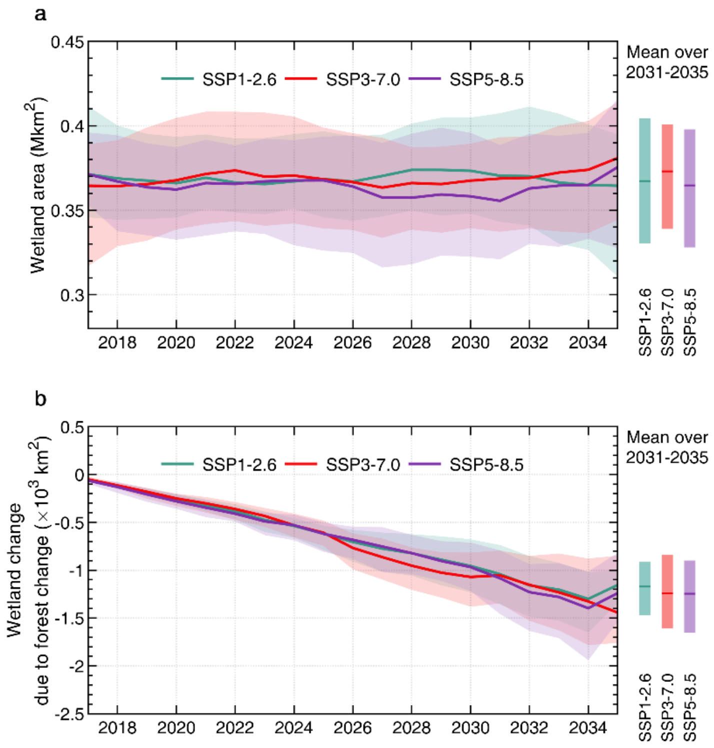

Supplementary Figure 18. Spatial distributions of mean annual forest area from 2000 to 2016 from NFI and two satellite-based forest cover products. Details about the three products are listed in Supplementary Table 3.

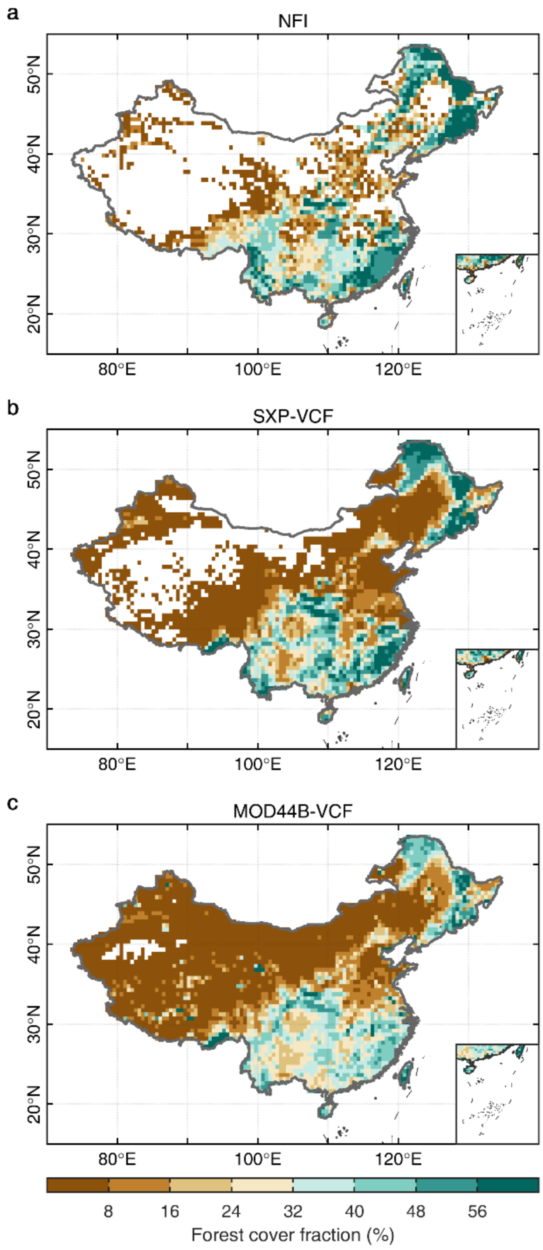

Supplementary Figure 19. Same as Fig. 1a and 1d, but for two satellite-based forest cover products.

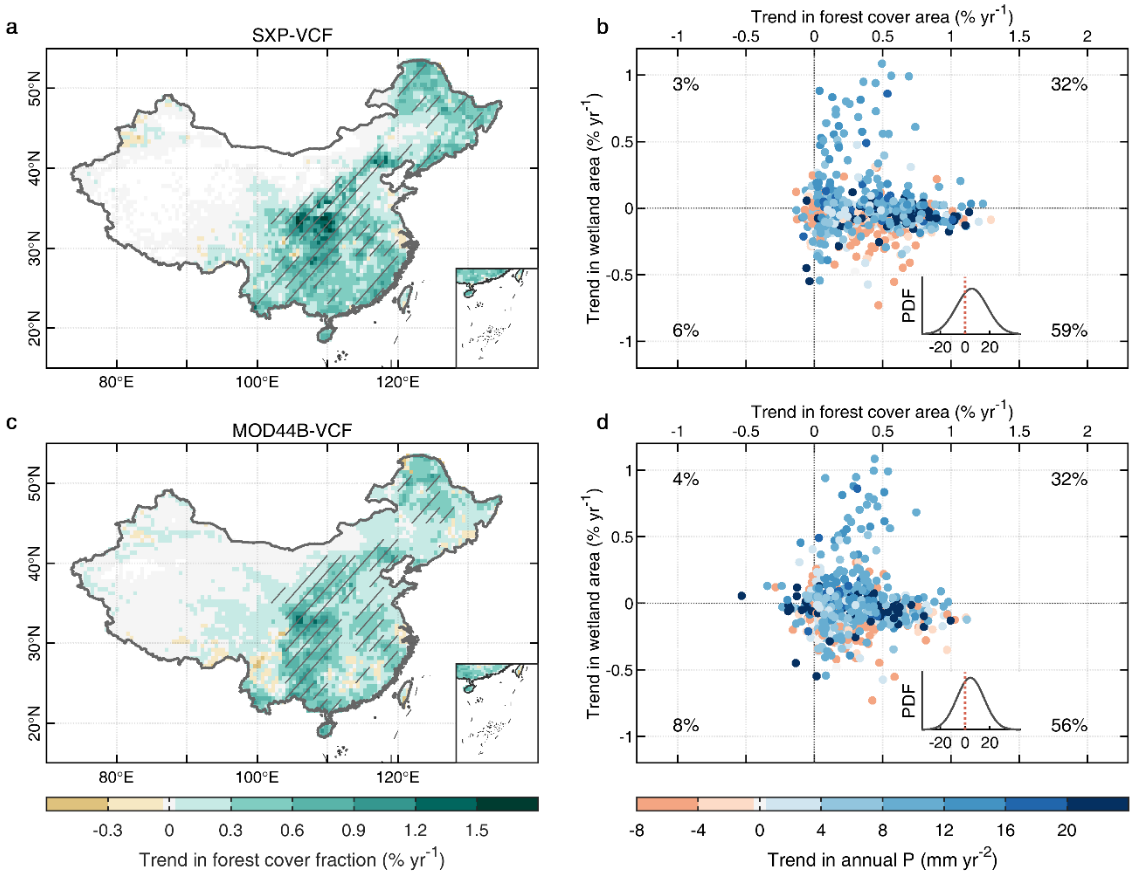

References

1 Song, X.-P. *et al.* Global land change from 1982 to 2016. *Nature* **560**, 639-643, (2018).

2 Li, Y. *et al.* Inconsistent estimates of forest cover change in China between 2000 and 2013 from multiple datasets: differences in parameters, spatial resolution, and definitions. *Sci. Rep.* **7**, 8748, (2017).

3 Hou, X. Vegetation atlas of China. *Chinese Academy of Science, the editorial board of vegetation map of China* (2001).
