# Supplementary material for: Trade-off between tree planting and wetland conservation in China
Source: Nat Commun. 2022 Apr 12;13:1967. doi: 10.1038/s41467-022-29616-7 (PMC9005732; doi:10.1038/s41467-022-29616-7)
Supplement: Supplementary file 3 — Reporting Summary [file 41467_2022_29616_MOESM3_ESM.pdf]

nature portfolio

Corresponding author(s): Shushi Peng
Last updated by author(s): Feb 9, 2022

Reporting Summary

Nature Portfolio wishes to improve the reproducibility of the work that we publish. This form provides structure for consistency and transparency in reporting. For further information on Nature Portfolio policies, see our Editorial Policies and the Editorial Policy Checklist.

Statistics

For all statistical analyses, confirm that the following items are present in the figure legend, table legend, main text, or Methods section.

n/a	Confirmed	
☐	☒	The exact sample size (*n*) for each experimental group/condition, given as a discrete number and unit of measurement
☒	☐	A statement on whether measurements were taken from distinct samples or whether the same sample was measured repeatedly
☐	☒	The statistical test(s) used AND whether they are one- or two-sided<br>*Only common tests should be described solely by name; describe more complex techniques in the Methods section.*
☒	☐	A description of all covariates tested
☒	☐	A description of any assumptions or corrections, such as tests of normality and adjustment for multiple comparisons
☐	☒	A full description of the statistical parameters including central tendency (e.g. means) or other basic estimates (e.g. regression coefficient) AND variation (e.g. standard deviation) or associated estimates of uncertainty (e.g. confidence intervals)
☐	☒	For null hypothesis testing, the test statistic (e.g. *F*, *t*, *r*) with confidence intervals, effect sizes, degrees of freedom and *P* value noted<br>*Give P values as exact values whenever suitable.*
☒	☐	For Bayesian analysis, information on the choice of priors and Markov chain Monte Carlo settings
☒	☐	For hierarchical and complex designs, identification of the appropriate level for tests and full reporting of outcomes
☒	☐	Estimates of effect sizes (e.g. Cohen's *d*, Pearson's *r*), indicating how they were calculated

Our web collection on statistics for biologists contains articles on many of the points above.

Software and code

Policy information about availability of computer code

Data collection	No software was used to collect data. All datasets are downloaded from the links noted in data availability.
Data analysis	The analyses and mapping were both performed using MATLAB (R2020a). The ORCHIDEE model were ran in the High-performance Computing Platform of Peking University. The code of ORCHIDEE-Hillslope can be accessed via https://forge.ipsl.jussieu.fr/orchidee/wiki/GroupActivities/CodeAvalaibilityPublication/ORCHIDEE-Hillslope-r6515. The code to simulate wetland area by TOPMODEL (Version v1.0) are publicly available on GitHub (https://github.com/yixixy/Wetland_simulation_by_TOPMODEL).

For manuscripts utilizing custom algorithms or software that are central to the research but not yet described in published literature, software must be made available to editors and reviewers. We strongly encourage code deposition in a community repository (e.g. GitHub). See the Nature Portfolio guidelines for submitting code & software for further information.

Data

Policy information about availability of data

All manuscripts must include a data availability statement. This statement should provide the following information, where applicable:

- Accession codes, unique identifiers, or web links for publicly available datasets
- A description of any restrictions on data availability
- For clinical datasets or third party data, please ensure that the statement adheres to our policy

All observation and model data that support the findings of this study are available as follows. The National Forest Inventory data are available from China's State Forestry Administration (http://www.forestry.gov.cn/). The GIEMS-2 dataset analyzed during the current study over China from 2000 to 2015 has been deposited on Zenodo (https://doi.org/10.5281/zenodo.5750962). The RFW data sets are available at https://doi.pangaea.de/10.1594/PANGAEA.892657. The HYDE v3.2 data set are available at https://easy.dans.knaw.nl/ui/datasets/id/easy-dataset:74467. The historical and future climate data from GSWP3-W5E5 and ISIMIP3b are obtained

from https://esg.pik-potsdam.de/search/isimip/. The protected wetland locations in China are obtained from http://www.zrbhq.cn/web/confirm.html. The shapefile data of basins at level 6 as classified by the global HydroBASINS database are available at https://www.hydrosheds.org/downloads.

Field-specific reporting

Please select the one below that is the best fit for your research. If you are not sure, read the appropriate sections before making your selection.

☐ Life sciences ☐ Behavioural & social sciences ☒ Ecological, evolutionary & environmental sciences

For a reference copy of the document with all sections, see nature.com/documents/nr-reporting-summary-flat.pdf

Ecological, evolutionary & environmental sciences study design

All studies must disclose on these points even when the disclosure is negative.

Study description	We employed satellite-derived inundation data and a process-based land surface model to investigate the impacts of historical (2000–2016) and near-term (2017–2035) afforestation on wetland dynamics in China.
Research sample	We used the gridded climate, land cover, and wetland extent data, which are all obtained from publicly available sources. All datasets include:<br>The National Forest Inventory data are obtained from China's State Forestry Administration (http://www.forestry.gov.cn/). The GIEMS-2 inundated data are obtained from Catherine Prigent, and can be downloaded at https://doi.org/10.5281/zenodo.5750962. The RFW data sets are available at https://doi.pangaea.de/10.1594/PANGAEA.892657. The HYDE v3.2 data set is available at https://easy.dans.knaw.nl/ui/datasets/id/easy-dataset:74467. The GSWP3-W5E5 and ISIMIP3b climate data are obtained from https://esg.pik-potsdam.de/search/isimip/. The protected wetland locations in China are obtained from http://www.zrbhq.cn/web/confirm.html. The shapefile of basins at level 6 as classified by the global HydroBASINS database are available at https://www.hydrosheds.org/downloads.
Sampling strategy	We used all samples to analyze the change in forest cover fraction and wetland extent in China. To clearly show the statistical results, the grid cells with no forest coverage, or no change in forest cover fraction, or a less than 1% wetland fraction from RFW are not shown in some figures.
Data collection	All data sets were downloaded from publicly available sources listed in the data availability statement in the main text.
Timing and spatial scale	The National Forest Inventory data have a 5-year temporal resolution and province-scale spatial resolution. The GIEMS-2 data have a monthly temporal resolution and $0.25° \times 0.25°$ spatial resolution. The RFW data are static and have a 15 arc-sec spatial resolution. The HYDE v3.2 data have a yearly temporal resolution and 5' spatial resolution. The GSWP3-W5E5 and ISIMIP3b climate data has a monthly temporal resolution and $0.5° \times 0.5°$ spatial resolution. The protected wetland locations and the shapefile of basins in China are static.
Data exclusions	We excluded grid cells with no forest coverage, or no change in forest cover fraction, or a less than 1% wetland fraction from RFW in some figures.
Reproducibility	Our analyses were based on public data sets and well-defined methods, and the results could be reliably reproduced.
Randomization	N/A
Blinding	Our results only used existing data, therefore blinding is not relevant to our study.

Did the study involve field work? ☐ Yes ☒ No

Reporting for specific materials, systems and methods

We require information from authors about some types of materials, experimental systems and methods used in many studies. Here, indicate whether each material, system or method listed is relevant to your study. If you are not sure if a list item applies to your research, read the appropriate section before selecting a response.

Materials & experimental systems

n/a	Involved in the study	
☒	☐	Antibodies
☒	☐	Eukaryotic cell lines
☒	☐	Palaeontology and archaeology
☒	☐	Animals and other organisms
☒	☐	Human research participants
☒	☐	Clinical data
☒	☐	Dual use research of concern

Methods

n/a	Involved in the study	
☒	☐	ChIP-seq
☒	☐	Flow cytometry
☒	☐	MRI-based neuroimaging
